# Safety of Lumason® (SonoVue®) in special populations and critically ill patients

**DOI:** 10.3389/fcvm.2023.1225654

**Published:** 2023-08-02

**Authors:** A. Filippone, M. A. Kirchin, J. Monteith, M. L. Storto, A. Spinazzi

**Affiliations:** ^1^ Global Medical & Regulatory Affairs, Bracco Imaging SpA, Milan, Italy; ^2^ Global Medical & Regulatory Affairs, Bracco Diagnostics Inc., Monroe, NJ, United States

**Keywords:** Lumason, SonoVue, ultrasound contrast agents, safety, echocardiography

## Abstract

Evidence for the safe use of Lumason® (SonoVue®), an ultrasound enhancing agent (UEA), in special patient populations is critical to enable healthcare professionals to make informed decisions concerning its use in such patients. Herein, we provide insight on the safety and tolerability of Lumason® in special patient populations. Findings are presented from clinical pharmacology studies conducted in patients with compromised cardiopulmonary conditions, from a retrospective study performed in critically ill patients, and from post-marketing surveillance data from over 20 years of market use of Lumason® (SonoVue®). No detrimental effects of Lumason® on cardiac electrophysiology were observed in patients with coronary artery disease (CAD), and no significant effects on pulmonary hemodynamics were noted in patients with pulmonary hypertension or congestive heart failure. Similarly, no effects on several assessments of pulmonary function (e.g., FVC) were observed in patients with chronic obstructive pulmonary disease (COPD), and no clinically meaningful changes in O_2_ saturation or other safety parameters were observed after administration of Lumason® to patients with diffuse interstitial pulmonary fibrosis (DIPF). The retrospective study of critically ill patients revealed no significant difference for in-hospital mortality between patients administered Lumason® for echocardiography versus those who had undergone echocardiography without contrast agent. Post-marketing surveillance revealed very low reporting rates (RR) for non-serious and serious adverse events and that serious hypersensitivity reactions were rare. These findings confirm that Lumason® is a safe and well tolerated UEA for use in special populations and critically ill patients.

## Introduction

Contrast-enhanced ultrasound (CEUS) is an increasingly accepted imaging modality, and numerous international society guidelines and position statements highlight the value of ultrasound enhancing agents (UEAs) in routine clinical practice for both cardiac and non-cardiac imaging (1, 2). Unfortunately, concern over the safety of UAEs was raised in the United States in 2007 when the Food and Drug Administration (FDA) placed a boxed warning on the labels of the UEAs, Optison™ (GE Healthcare Inc., Marlborough, MA) and Definity® (Lantheus Medical Imaging Inc., N. Billerica, MA) contraindicating their use in patients with unstable cardiopulmonary status and pulmonary hypertension and mandating a 30 min monitoring period for all patients receiving a UEA. In light of new safety information from published studies (3–23), the FDA has since revised the labeling for UEAs, downgrading the boxed warning from contraindications to warnings and removing the requirement for a 30 min monitoring period. Currently, the only contraindication in the labeling of UEAs approved in the United States is “known or suspected hypersensitivity to the gas or other agent components”, while the boxed warning refers only to the uncommon occurrence of serious cardiopulmonary reactions, mostly within 30 min of UEA administration, and the need for resuscitation equipment and trained personnel to be readily available.

Lumason® [Bracco Diagnostics Inc., Monroe, NJ (known as SonoVue® outside the United States)] is a second-generation UEA composed of sulfur hexafluoride (SF6) microbubbles which was approved by the FDA in 2014 for intravenous administration in adults and pediatric patients to opacify the left ventricle and improve endocardial border delineation and to characterize focal liver lesions. Precise wording on the Lumason USA Package insert states that it is indicated for use in in echocardiography “*to opacify the left ventricular chamber and to improve the delineation of the left ventricular endocardial border in adult and pediatric patients with suboptimal echocardiograms*” (24). This is similar to that for SonoVue® in Europe which states that it “*is indicated for use in adult patients with suspected or established cardiovascular disease to provide opacification of cardiac chambers and enhance left ventricular endocardial border delineation*” (25). It is also approved in the USA and elsewhere for intravesical administration in pediatric patients with known or suspected vesicoureteral reflux (24, 25). Outside of the United States, Lumason® (SonoVue®) is additionally approved for use in adults for macrovascular applications and for characterization of breast lesions. The target patient population for Lumason® is therefore very diverse in terms of demographics, disease state, and pathology, and includes critically ill patients and compromised patients with reduced cardiopulmonary function who might benefit specifically from the improved image quality provided by UEAs (26–28).

As part of its clinical development program, clinical pharmacology studies aimed at evaluating Lumason® pharmacokinetics and pharmacodynamics as well as the impact of intrinsic factors on exposure were conducted in patients with coronary artery disease (CAD), congestive heart failure, moderate to severe chronic obstructive pulmonary disease (COPD), and diffuse interstitial pulmonary fibrosis (DIPF). Here below we present the principal findings from these studies. Also presented are the results of a retrospective study to assess the safety of Lumason® in critically ill patients in whom the feasibility of transthoracic echocardiographic imaging is often limited due to a complex and frequently dynamic clinical profile or because of other extrinsic factors such as patients being uncooperative, hyperinflated lungs due to mechanical ventilation, lung disease, subcutaneous emphysema, surgical incisions, chest tubes, and bandages (26–28). Finally, to complete the safety overview of Lumason®, post-marketing surveillance data are presented from over 20 years of market use of this UEA.

In providing a comprehensive assessment of the safety and tolerability of Lumason® in key patient populations, we aim to demonstrate the practical utility of this UEA for clinical use across the range of compromised and critically ill patients for whom administration of a UEA is considered necessary.

## Methods and materials

### Clinical pharmacology studies in special patient populations

A summary of pharmacokinetic studies in special patient populations is presented in Table 1. Complete descriptions of the methods employed for these studies are presented in Appendix 1.

**Table 1:** Summary of pharmacokinetic studies in special patient populations.

Study	Study design	Evaluation	Results
Effects on ventricular repolarization	Placebo-controlled prospective safety studies of continuous ECG monitoring in patients with coronary artery disease • 3-way crossover study of placebo and 2 doses of Lumason® (0.1 and 0.5 ml/kg) • 4-way crossover study to evaluate cardiac electrophysiology during insonation of the heart at low (0.4–0.5) and high (1.5–1.6) mechanical index	Continuous 12-lead ECG collected from 3 h pre-dose to 12 h post-dose following each administration of study agent	No detrimental effects of Lumason® on cardiac electrophysiology were observed
Patients with pulmonary hypertension or congestive heart failure	Single center, randomized placebo-controlled studies to evaluate the effects of iv bolus injections of Lumason® on pulmonary hemodynamics and cardiac function in patients with • normal or elevated baseline MPAP scheduled for right heart catheterization as part of routine evaluation • congestive heart failure	PVR, MPAP, PCWP monitored by right heart catheterization predose and up to 10 min post-dose. Cardiac function and O2 saturation were measured	No significant effects on pulmonary hemodynamics after Lumason® or placebo were observed and no differences between 2-ml and 4-ml doses were seen
Patients with COPD	Single-center, single-blind, crossover, placebo-controlled study of 4 ml bolus iv Lumason® injection in patients with moderate to severe COPD and forced expiratory volume (FEV1) <70%	Pulmonary function (FVC, FEV1 and FEF_25−75%_) measured at time points up to 5 h post-dose	No effect of Lumason® on pulmonary function tests, O_2_ saturation, ECG, or laboratory tests was observed
Patients with DIPF	Single-center, phase I study to evaluate the pharmacokinetics and safety of a single iv bolus injection of Lumason 0.3 ml/kg in patients with DIPF	O_2_ saturation through 1-hour post-dose	No changes or clinically meaningful trends observed in O_2_ saturation or other safety parameters

PVR, pulmonary vascular resistance; MPAP, pulmonary arterial pressure; PCWP, pulmonary capillary wedge pressure; FEV, forced expiratory volume; FVC, forced vital capacity; FEF, forced mid-expiratory flow; COPD, chronic obstructive pulmonary disease; DIPF, diffuse interstitial pulmonary fibrosis.

#### Assessment of potential effects on ventricular repolarization in patients with coronary artery disease

A delay in cardiac repolarization creates an electrophysiological environment that favors the development of cardiac arrhythmias. To evaluate the potential risk of cardiac arrhythmias, assessment of the risk of QT prolongation is now a standard regulatory requirement for the clinical development of any new drug entity, including UEAs (29).

Two prospective studies were conducted to assess the effects of Lumason® on ventricular repolarization in patients with documented CAD undergoing echocardiography. In the first study (a single-blind, placebo-controlled, randomized, 3-way crossover study), each subject received two bolus injections of Lumason® at doses of 0.1 and 0.5 ml/kg, and one injection of placebo (0.9% normal physiological saline) in a sequence determined by randomization. In the subsequent study (a single-blind, placebo-controlled, randomized, 4-way crossover study), each subject received 2 injections of Lumason® at the same dose of 0.1 ml/kg and 2 injections of placebo (0.9% normal physiological saline), again in randomized fashion. Two different MI settings (low/medium: 0.4–0.5 and high: 1.5–1.6) with continuous insonation were used for echocardiography with each of the Lumason® and placebo injections.

In both studies, two-dimensional (2D) echocardiography of the left ventricle was performed prior to the administration of study agent and for continuous intervals (5 min in the 3-way study and 20 min in the 4-way study) following study agent administration. Continuous 12-lead ECG data were collected from 3 h predose to 12 h postdose following each administration of study agent. Other safety assessments performed included evaluation of clinical and laboratory markers for potential microembolism and monitoring for the occurrence of adverse events.

#### Assessment of potential effects of Lumason® on pulmonary hemodynamics and cardiac function

To be effective, UEA microbubbles should pass through the pulmonary vascular bed without obstructing capillary flow. A possible consequence of pulmonary capillary obstruction is a decrease in oxygen saturation leading to an increase in pulmonary arterial pressure and pulmonary vascular resistance, which in turn can cause a cardio-depressive or negative inotropic effect. In patients with pre-existing pulmonary hypertension, occlusion of a portion of the pulmonary microvasculature might lead to hemodynamic compromise (30). To avoid these potentially harmful effects, UEA microbubbles should not be larger than 10 μm in diameter. Lumason® microbubbles are very small in size, with a mean diameter ranging between 1.5 and 2.5 µm and 99% having a diameter ≤10 µm. To date, no negative effects on oxygen saturation or negative inotropic effects have been reported.

Nevertheless, to investigate the possible effects of Lumason® on pulmonary hemodynamics and cardiac function, studies were performed in patients with and without pulmonary hypertension and in patients with congestive heart failure referred for right cardiac catheterization. Right heart catheterization is the most reliable and accurate method for the detection and diagnosis of pulmonary hypertension and for monitoring treatment effects on pulmonary pressures in patients with pulmonary hypertension. It allows for measurement and analysis of the right heart, pulmonary artery and pulmonary capillary wedge pressure, measurement of cardiac output, screening for intra-cardiac shunts, temporary ventricular pacing, assessment and treatment of arrhythmias and cardiac biopsy.

##### Patients with pulmonary hypertension

A multi-center, double-blind, randomized, placebo-controlled, intra-subject crossover study was performed to evaluate the effect of intravenous bolus injections of Lumason® in comparison with placebo on pulmonary hemodynamics in patients with normal (<25 mmHg; normal group) or elevated (≥25 mmHg; hypertension group) baseline mean pulmonary arterial pressure who were scheduled to undergo right heart catheterization as part of their routine clinical evaluation. Each patient received a single intravenous bolus injection of 4.8 ml Lumason® and a single bolus injection of 4.8 ml placebo (0.9% normal physiological saline) in randomized fashion. Both injections were followed by a 5 ml saline flush. All patients underwent standard right heart catheterization using a Swan-Ganz catheter from a jugular or femoral venous access. Multiple pulmonary hemodynamic and cardiac parameters were measured before and after each injection. These parameters included pulmonary vascular resistance (PVR), mean pulmonary artery pressures (MPAP), pulmonary capillary wedge pressure (PCWP), O_2_ saturation, heart rate, and cardiac output.

##### Patients with congestive heart failure

The study in patients with congestive heart failure [NYHA (New York Heart Association) class II-III] and ejection fraction (EF) <45%, was a placebo-controlled, single center, study in which patients were randomized to one of two study arms (31). Patients in one arm received two bolus injections of Lumason® (2.0 and 4.0 ml) and two injections of placebo (2.0 and 4.0 ml of 0.9% physiological saline) according to a four-dose sequence randomization in which Lumason® and placebo were administered alternately. Patients in the other arm received two injections of placebo (2.0 and 4.0 ml) only according to a two-dose sequence randomization. The effects of Lumason® compared with placebo on pulmonary hemodynamics and cardiac function were monitored before the first administration and again at 30 s, and 2, 4, 6, 10 min after each administration.

#### Assessment of oxygen saturation and pulmonary function in patients with moderate to severe COPD

The gas contained in microbubble based UEA agents such as Lumason® is eliminated through the lungs with expired air. To determine whether Lumason® impacts pulmonary function, oxygen saturation or the persistence of microbubbles in the blood of patients with moderate or severe COPD, a single-center, single-blind, crossover, placebo-controlled, intravenous fixed-dose study was performed (32). Each patient received a single injection of 4 ml of Lumason® and a single injection of 4 ml of placebo in randomized fashion in two sessions separated by 48–72 h. Pulmonary function testing of forced vital capacity (FVC), forced expiratory volume (FEV_1_), and forced mid-expiratory flow (FEF_25%−75%_) was performed at baseline and at several timepoints up to 5 h after each injection.

#### Assessment of pharmacokinetics and oxygen saturation in patients with DIPF

Diffuse interstitial pulmonary fibrosis (DIPF) is another condition in which patients have reduced alveolar function which might potentially impact the pulmonary elimination of gas. An open-label, single-dose, single-center study was conducted to evaluate the pharmacokinetics and safety of Lumason® at a dose of 0.3 ml/kg (ten-fold higher than the approved dose of 0.03 ml/kg) in patients with reduced alveolar function due to mild to severe DIPF compared to patients with normal alveolar function. Blood was collected for determination of SF6 concentration at one minute before injection and at multiple postdose timepoints up to approximately 2 h after Lumason® injection. Concentrations of SF6 in expired air were determined at one to two minutes before injection and at multiple timepoints postdose. Standard pharmacokinetic parameters determined included area under the blood-concentration time curve, maximum blood SF6 concentration, time of maximum blood SF6 concentration, apparent total body clearance, terminal elimination half-life, and apparent volume of distribution at steady-state.

### Safety studies

#### Safety of Lumason® in critically ill patients

It is estimated that 10%–15% of routine echocardiograms have suboptimal border definition and that this percentage increases to 25%–30% in critically ill patients (28). Given that UEAs are indicated to improve endocardial border delineation in patients with suboptimal echocardiograms, it is essential that the safety of UEAs in critically ill patients is assessed. A retrospective non-interventional post-authorization study compared in-hospital mortality (defined as death within the same day or the following calendar day of an echocardiography procedure) between critically ill patients who had undergone contrast echocardiography with Lumason® and critically ill patients who had undergone echocardiography without the use of a UEA. Adverse events were collected whenever such information was available in patients’ medical records.

#### Safety of Lumason from post-marketing surveillance

The total number of serious and non-serious adverse events that were spontaneously reported during market use between 1 April 2001 and 28 February 2023 which were considered possibly or probably related to the administration of Lumason® were collected.

## Results

### Assessment of potential effects on ventricular repolarization in patients with coronary artery disease

One hundred and one patients with documented CAD were included: 48 patients (24 males, 24 females, mean age 62.8 years, range 45–79 years) completed the 3-way crossover study, and 53 patients (28 males, 25 females, mean age 63.1 years, range 45–79 years) completed the 4-way crossover study. No detrimental effects of Lumason® on cardiac electrophysiology were observed.

In the 3-way crossover study, the primary analysis of maximum mean increase from baseline for corrected individualized QT (QTcI) values confirmed that there was no significant difference between placebo and Lumason®. No dose or time dependency was observed with respect to mean QTcI intervals at baseline. The mean maximum increase in QTcI values from baseline (maximum postdose measurement) were comparable for placebo and Lumason®: placebo: 18.4 msec, Lumason® 0.1 ml/kg: 16.8 msec, and Lumason 0.5 ml/kg: 17.5 msec (Table 2).

**Table 2:** Analysis of maximum increase from baseline in QTcI interval (msec) within 1-h postdose.

Parameter	Placebo	Lumason 0.1 ml/kg	Lumason 0.5 ml/kg
	(*N* = 48)	(*N* = 48)	(*N* = 48)
Baseline^a^			
Mean (SD)	404.6 (22.7)	403.9 (24.5)	401.8 (23.0)
Median	404.5	399.0	401.0
Range (Min—Max)	362–456	366–465	360–451
Maximum Postdose Value^b^			
Mean (SD)	422.9 (21.9)	420.7 (26.4)	419.3 (23.0)
Median	420.5	414.0	417.0
Range (Min—Max)	385–475	373–486	377–468
Maximum Increase from Baseline^c^			
LS Mean Change (SE)	18.4 (1.3)	16.8 (1.3)	17.5 (1.3)
95% CI for LS Mean Change	(15.9, 20.8)	(14.3, 19.2)	(15.0, 20.0)
Lumason—Placebo			
Difference in LS Mean Change (SE)		–1.60 (1.60)	–0.85 (1.60)
95% CI for Difference of LS Mean Change		(–4.79, 1.58)	(–4.04, 2.33)
Lumason 0.5 ml/kg—Lumason 0.1 ml/kg			
Difference in LS Mean Change (SE)		0.75 (1.60)	
95% CI for Difference of LS Mean Change		(–2.43, 3.93)	

QTc Interval Normal Range: 320–440 msec.

QTcI, Individual subject corrected QT interval; SD, Standard deviation; SE, Standard error; LS, Least squares; CI, Confidence interval; Min, minimum; Max, maximum.

^a^ Baseline is the mean of all technically adequate recorded values from 3 h predose to immediately predose.

^b^ Postdose value from +1 min to +1 h where the maximum increase from baseline occurred.

^c^ Based on an ANOVA model including dose and period as fixed effects and subject as random effect.

Similar results were observed in the 4-way crossover study; baseline and maximum post-dose mean QTcI values were comparable among the 4 treatments (Lumason® 0.1 ml/kg at MI 0.4 and 1.5; placebo 0.1 ml/kg at MI 0.4 and 1.5). There were no clinically meaningful differences among the 4 treatment groups with respect to mean QTcI interval values at baseline or at the maximum postdose measurement (Table 3). There were no statistically significant differences in maximum increase from baseline of QTcI between placebo and Lumason at low or high MI. Furthermore, there was no evidence that administration of Lumason® is associated with an increased risk of microembolism. No serious or other non-serious adverse events were reported during these studies.

**Table 3:** Analysis of maximum increase from baseline in QTcI interval (msec) within 1-h postdose.

Parameter	Placebo MI 0.4	Placebo MI 1.5	Lumason MI 0.4	Lumason MI 1.5
	(*N* = 50)	(*N* = 50)	(*N* = 50)	(*N* = 50)
Baseline^a^				
Mean (SD)	403.0 (24.0)	402.4 (24.1)	401.1 (24.7)	402.1 (23.8)
Median	400.0	397.0	399.5	400.0
Min—Max	358–454	360–460	349–454	363–458
Maximum Postdose Value^b^				
Mean (SD)	421.4 (26.8)	420.0 (26.5)	420.0 (26.4)	418.8 (25.7)
Median	419.0	418.0	420.5	415.0
Min—Max	364–487	378–486	365–475	375–480
Maximum Increase from Baseline^c^				
LS Mean Change (SE)	18.5 (1.3)	17.7 (1.3)	18.9 (1.3)	16.7 (1.3)
95% CI for LS Mean Change	(15.8, 21.1)	(15.0, 20.3)	(16.3, 21.6)	(14.0, 19.3)
Placebo MI 0.4– Lumason MI 0.4				
Difference in LS Mean Change (SE)			−0.49 (1.59)	
95% CI for Difference of LS Mean Change			(−3.63, 2.66)	
Placebo MI 1.5– Lumason MI 1.5				
Difference in LS Mean Change (SE)				1.03 (1.59)
95% CI for Difference of LS Mean Change				(−2.11, 4.18)

QTc Interval Normal Range: 320-440 msec.

Only 50 of 53 enrolled patients were evaluated. Three patients were excluded from the analysis population due to technically inadequate ECG data.

MI, Mechanical Index; QTcI, Individual subject corrected QT interval; SD, Standard deviation; SE, Standard error; LS, Least squares; CI, Confidence interval; Min, Minimum; Max, Maximum.

^a^ Baseline is the mean of all technically adequate recorded values from 3 h predose to immediately predose.

^b^ Postdose value from +1 min to +1 h where the maximum increase from baseline occurred.

^c^ Based on an ANOVA model including treatment and period as fixed effects and subject as random effect.

### Assessment of potential effects of Lumason® on pulmonary hemodynamics and cardiac function

#### Patients with pulmonary hypertension

Thirty-six patients (18 with hypertension [13 males, 5 females, mean age 58.0 years, range 35–86 years; 10 assigned to placebo/Lumason® and 8 to Lumason®/placebo] and 18 without hypertension [12 males, 6 females, mean age 57.2 years, range 29–73 years; 8 assigned to placebo/Lumason® and 10 to Lumason®/placebo]) were included in the analysis.

**Figure 1:**
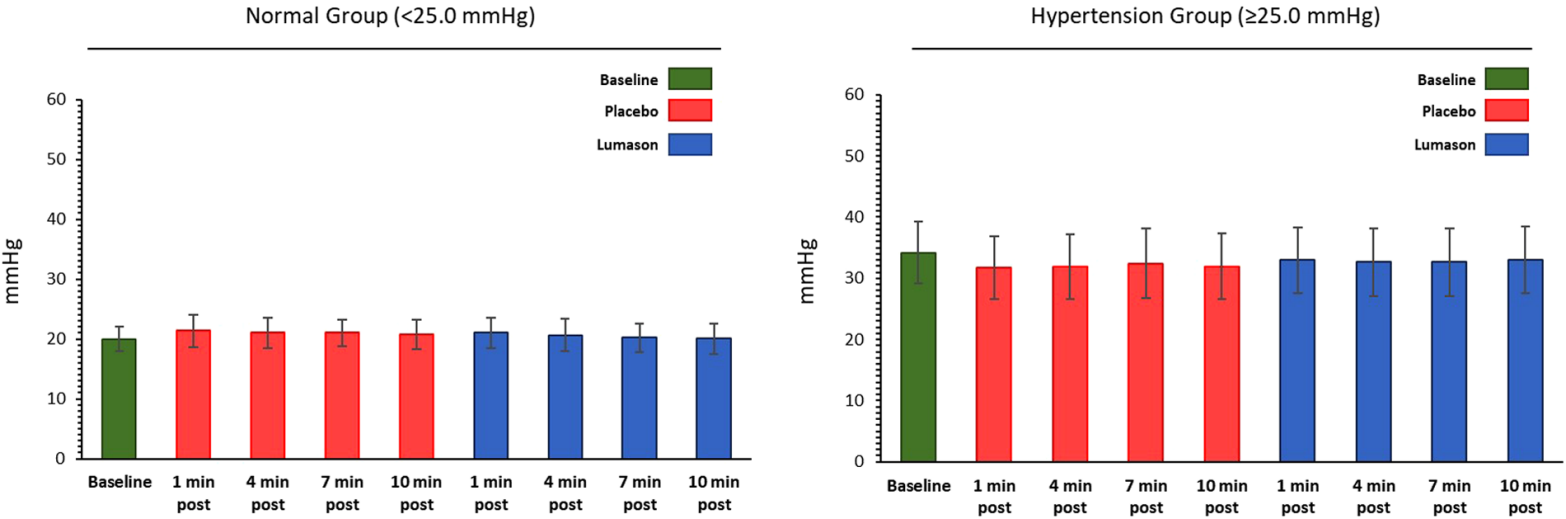
Hemodynamic parameter: mean pulmonary artery pressure (mmHg)—mean values at each time point by group.

**Figure 2:**
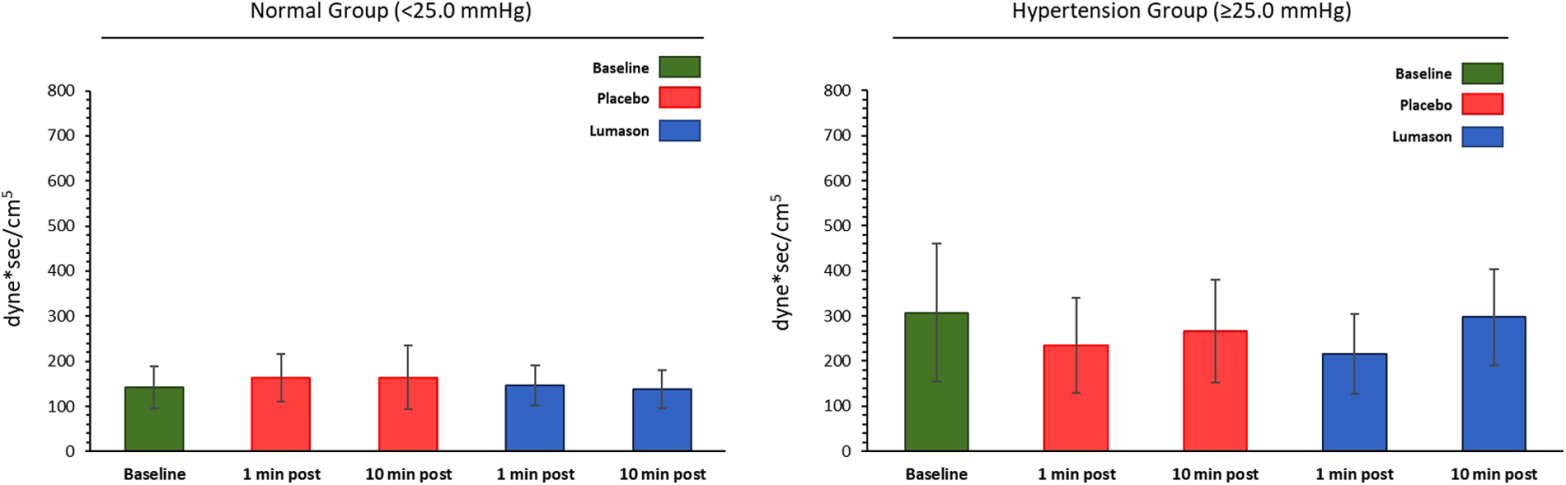
Hemodynamic parameter: pulmonary vascular resistance (dyne*sec/cm^5^)—mean values at each time point by group.

The administration of Lumason® did not have any clinically relevant effect on pulmonary hemodynamic parameters in patients with elevated or normal mean pulmonary artery pressure. Mean changes from baseline were small for all hemodynamic parameters across all post-dose time points and no significant differences were noted between Lumason® and placebo in either the hypertension or the normal groups (mean differences ranged between 0.2 and 2 for most parameters; Table 4, Figure 1). This result was not influenced by the order of administration of Lumason® and placebo. A slightly higher variability, especially in patients with elevated baseline pulmonary arterial pressure, was observed for mean changes of pulmonary vascular resistance with both Lumason® and placebo (Figure 2).

**Table 4:** Pulmonary hemodynamic parameters—mean change (sd) from baseline at maximum increase and at maximum decrease.

Parameter	Hypertension Group (*N* = 18)					Normal Group (*N* = 18)				
	Baseline	Lumason		Placebo		Baseline	Lumason		Placebo	
		Max Dec	Max Inc	Max Dec	Max Inc		Max Dec	Max Inc	Max Dec	Max Inc
PAPs (mmHg)	*N* = 18	*N* = 16	*N* = 11	*N* = 15	*N* = 9	*N* = 18	*N* = 11	*N* = 14	*N* = 10	*N* = 16
Mean (SD)	50.50 (17.581)	−6.719 (5.174)	2.818 (2.000)	−6.367 (5.034)	4.111 (3.008)	30.08 (6.001)	−3.500 (3.391)	3.071 (0.917)	−3.400 (3.486)	4.188 (3.728)
95% CIs (LL, UL)		(−9.476, −3.962)	(1.415, 4.222)	(−9.154, −3.579)	(1.799, 6.423)		(−5.778, −1.222)	(2.542, 3.601)	(−5.894, −0.906)	(2.201, 6.174)
PAPd (mmHg)	*N* = 18	*N* = 16	*N* = 11	*N* = 16	*N* = 8	*N* = 18	*N* = 11	*N* = 17	*N* = 9	*N* = 17
Mean (SD)	24.11 (7.177)	−6.813 (6.516)	3.000 (1.225)	−5.938 (4.701)	2.813 (1.907)	11.72 (3.750)	−2.364 (2.820)	2.853 (3.220)	−2.389 (2.583)	3.324 (2.910)
95% CIs (LL, UL)		(−10.285, −3.340)	(2.177, 3.823)	(−8.442, −3.433)	(1.218, 4.407)		(−4.258, −0.469)	(1.197, 4.508)	(−4.375, −0.403)	(1.827, 4.820)
MPAP (mmHg)	*N* = 18	*N* = 11	*N* = 11	*N* = 13	*N* = 8	*N* = 18	*N* = 9	*N* = 14	*N* = 9	*N* = 17
Mean (SD)	34.19 (10.072)	−5.636 (2.703)	2.773 (2.504)	−5.731 (3.251)	3.375 (3.137)	20.00 (4.040)	−2.222 (2.265)	2.429 (1.719)	−1.778 (1.417)	2.735 (1.697)
95% CIs (LL, UL)		(−7.452, −3.821)	(1.091, 4.455)	(−7.695, −3.766)	(0.753, 5.997)		(−3.964, −0.481)	(1.436, 3.421)	(−2.867, −0.689)	(1.863, 3.608)
PCWP (mmHg)	*N* = 18	*N* = 12	*N* = 10	*N* = 9	*N* = 9	*N* = 18	*N* = 7	*N* = 9	*N* = 10	*N* = 7
Mean (SD)	18.8 (7.15)	−3.500 (3.289)	4.400 (4.061)	−4.556 (3.712)	5.444 (5.411)	12.3 (4.60)	−3.000 (2.236)	3.444 (2.242)	−2.000 (1.414)	3.286 (2.928)
95% CIs (LL, UL)		(−5.590, −1.410)	(1.495, 7.305)	(−7.409, −1.702)	(1.285, 9.604)		(−5.068, −0.932)	(1.721, 5.168)	(−3.012, −0.988)	(0.578, 5.993)
Qp (litres/min)	*N* = 18	*N* = 13	*N* = 9	*N* = 13	*N* = 9	*N* = 18	*N* = 8	*N* = 14	*N* = 10	*N* = 12
Mean (SD)	4.926 (2.1058)	−0.645 (0.643)	0.560 (0.333)	−0.728 (0.465)	0.847 (0.512)	4.763 (1.4543)	−0.449 (0.442)	0.729 (0.742)	−0.503 (0.520)	0.559 (0.540)
95% CIs (LL, UL)		(−1.033, −0.256)	(0.304, 0.816)	(−1.118, −0.339)	(0.453, 1.240)		(−0.818, −0.079)	(0.300, 1.157)	(−0.875, −0.131)	(0.216, 0.903)
Heart Rate (bpm)	*N* = 18	*N* = 10	*N* = 8	*N* = 9	*N* = 9	*N* = 18	*N* = 9	*N* = 9	*N* = 8	*N* = 9
Mean (SD)	80.1 (8.71)	−6.000 (5.375)	5.500 (6.782)	−8.444 (8.233)	5.000 (5.408)	77.7 (17.08)	−3.444 (2.651)	4.667 (7.858)	−3.250 (4.803)	4.556 (3.877)
95% CIs (LL, UL)		(−9.845, −2.155)	(−0.170, 11.170)	(−14.773, −2.116)	(0.843, 9.157)		(−5.482, −1.407)	(−1.374, 10.707)	(−7.266, 0.766)	(1.576, 7.535)
Stroke Volume (mL)	*N* = 18	*N* = 13	*N* = 11	*N* = 14	*N* = 10	*N* = 18	*N* = 7	*N* = 15	*N* = 11	*N* = 11
Mean (SD)	62.365 (27.201)	−7.990 (7.717)	6.536 (5.787)	−9.363 (7.861)	12.944 (14.437)	62.322 (18.749)	−5.492 (4.104)	8.680 (9.415)	−6.624 (7.501)	7.614 (5.873)
95% CIs (LL, UL)		(−12.653, −3.326)	(2.648, 10.424)	(−13.902, −4.824)	(2.617, 23.271)		(−9.288, −1.697)	(3.466, 13.894)	(−11.663, −1.585)	(3.669, 11.559)
PVR (dyne x s/cm^5^)	*N* = 18	*N* = 11	*N* = 12	*N* = 16	*N* = 9	*N* = 18	*N* = 11	*N* = 11	*N* = 10	*N* = 14
Mean (SD)	307.19 (307.63)	−138.515 (169.020)	78.254 (68.614)	−121.318 (220.423)	74.843 (74.211)	142.71 (93.61)	−47.834 (40.484)	43.119 (39.393)	−40.815 (36.466)	66.246 (73.625)
95% CIs (LL, UL)		(−252.064, −24.966)	(34.659, 121.849)	(−238.773, −3.862)	(17.799, 131.887)		(−75.032, −20.636)	(16.654, 69.583)	(−66.901, −14.729)	(23.737, 108.756)

The number of subjects with increases are included in the summary for maximum increase and those with decreases are included in the summary for maximum decrease; subjects with no change are not included.

Max Inc, Mean change from baseline at maximum increase; Max Dec, Mean change from baseline at maximum decrease; LL, Lower Limit; UL, Upper Limit.

Assessment of other safety parameters including vital signs, ECG, and laboratory tests did not reveal significant differences from baseline in patients in either the hypertension or normal group. No serious adverse events were reported.

#### Patients with congestive heart failure

Overall, 19 patients were included. Thirteen patients (12 males, 1 female, mean age 63.6 years, range 40–76 years) received two doses of Lumason® and two doses of placebo, while 6 patients (6 males, mean age 61.5 years, range 53–70 years) received two doses of placebo only. Baseline pulmonary hypertension (defined as PAPs >30 mmHg or PAPd >15 mmHg) was present in 11 patients in the Lumason® group and 5 patients in the control group.

**Figure 3:**
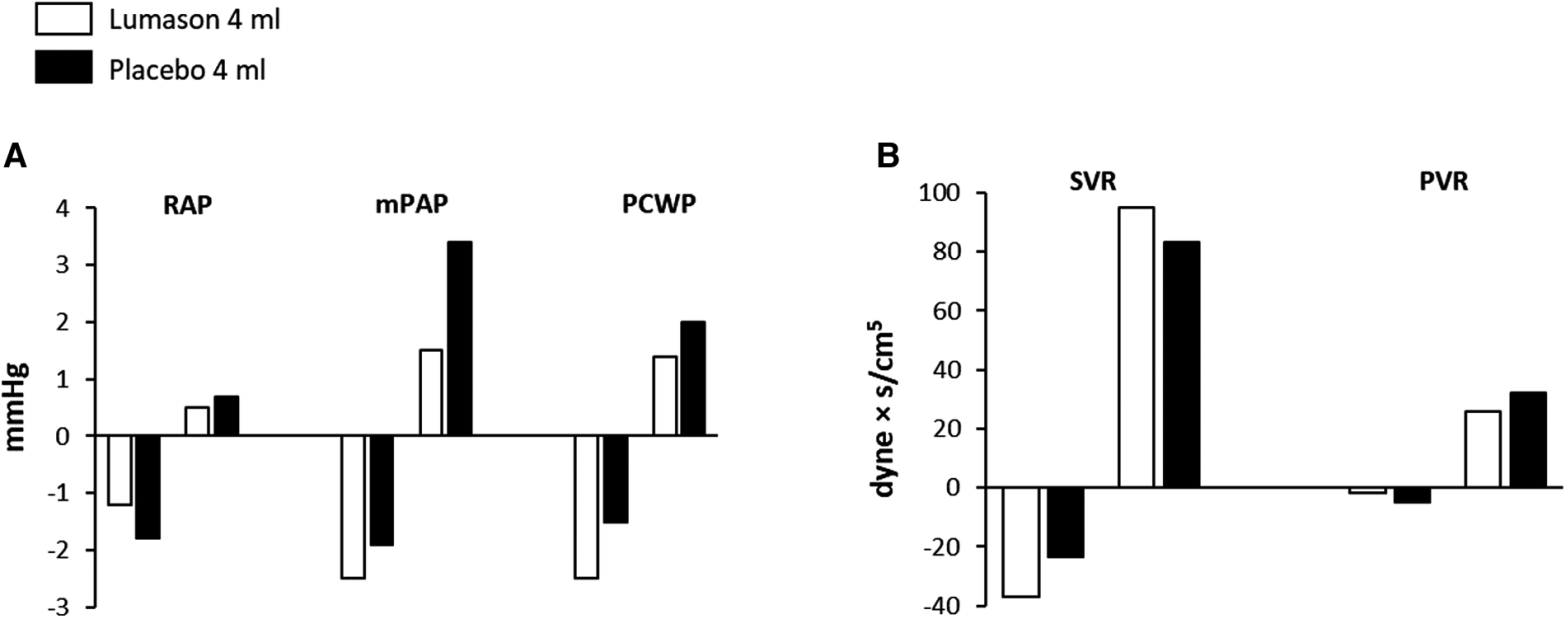
Effects of Lumason® administration on pulmonary and cardiac hemodynamics in patients with congestive heart failure. Mean deviation from baseline of the hemodynamic parameters (**A**) and systemic and pulmonary vascular resistance (**B**) measured after administration of Lumason and placebo. RAP, right atrial pressure; mPAP, mean pulmonary arterial pressure; PCWP, pulmonary capillary wedge pressure; SVC, systemic vascular resistance; PVR, pulmonary vascular resistance.

Baseline pulmonary hemodynamic, cardiac function, and oxygen saturation values were comparable for the two study groups. No differences were observed between Lumason® and placebo, nor between Lumason® 2 ml and Lumason® 4 ml within the Lumason® plus placebo arm. No differences were observed between the Lumason® plus placebo arm and the placebo only arm. No evidence of any trends over time for any of the hemodynamic parameters related to pulmonary circulation was observed in either study arm (Figure 3).

No changes in physical status at the 24 h follow-up examination were reported in any patient in either study arm. Differences in blood pressure and ECG pattern from screening to follow-up were not clinically significant. No serious adverse events were reported.

#### Assessment of oxygen saturation and pulmonary function in patients with moderate to severe COPD

Twelve patients (8 males, 4 females, mean age: 67.8 years, range: 50–84 years) were included. Six patients had moderate COPD (FEV_1_ = 51% to 69%), and 6 had severe COPD (FEV_1_ ≤ 50%).

Mean changes from baseline in oxygen saturation ranged from 21.3% to 0.9% after Lumason® and from 21.3% to 1.0% after placebo. No clinically significant changes from baseline in oxygen saturation were observed for either study agent at any post-injection time point. Table 5 presents mean changes from baseline in FEV_1_, FVC, and FEF_25%–75%_ at various post-injection time points in all patients. Changes in the mean values of pulmonary function parameters after administration of Lumason® and placebo were similar across the different time points. Mean and individual decreases from baseline in FEV_1_, FVC, FEF_25%−75%_ and oxygen saturation were observed after both Lumason® and placebo injection. Mean differences between Lumason® and placebo were small, and no consistent trends were observed across post-injection time points. No statistically significant differences between Lumason® and placebo were noted in terms of changes from baseline for pulmonary function parameters. No serious adverse events were reported.

**Table 5:** Lung function tests: change from baseline in FEV_1_, FVC, and FEF_25%–75%_ in patients with moderate or severe COPD following administration of Lumason®.

	Change from baseline post-injection at:	Lumason®—Placebo		
		Mean	SD	95% CI^b^
FEV_1_(ml)	1 min	1.9	193.54	−121.1, 124.9
	3 min	25.2	255.41	−137.1, 187.4
	9 min	−60.9	174.83	−172.0, 50.2
	10 min	−34.6	146.64	−127.8, 58.6
	11 min	−42.4	199.51	−169.2, 84.3
FVC (ml)	9 min	−103.7	278.22	−280.4, 73.1
	10 min	−73.9	265.94	−242.9, 95.1
	11 min	−97.0	359.80	−325.6, 131.6
FEF_25%–75%_ (L/s)	9 min	10.2	75.23	−37.6, 58.0
	10 min	5.6	125.44	−74.1, 85.3
	11 min	12.4	140.24	−76.7, 101.5

*n* = 12 for all assessments.

Taken from Reference (31).

FEV_1_, indicates forced expiratory volume; FVC, forced vital capacity; FEF, forced mid-expiratory flow; CI, confidence interval.

^a^ Difference between Lumason® (SonoVue™) and placebo in the change from baseline in FEV_1_, FVC, or FEF_25%–75%._

^b^ Based on a comparison between Lumason® (SonoVue™) and placebo in the change from baseline in FEV_1_ by using a paired *t-*test.

#### Assessment of pharmacokinetics and oxygen saturation in patients with DIPF

The study included 13 patients (8 males, 5 females, mean age: 55.6 years, range: 36–80 years) with known DIPF. Impairment of pulmonary function was rated on a 5-point scale as mild (3 patients), mild/moderate (4 patients), moderate (4 patients), moderate/severe (1 patient) or severe (1 patient). Pharmacokinetic parameters were estimated in only 12 patients as the dose administered to one patient was not recorded accurately at the time of administration.

Table 6 compares pharmacokinetic parameters for SF6 determined in patients with pulmonary impairment and healthy subjects who were administered Lumason® at a dose of 0.3 ml/kg. Higher mean *C*_max_ estimates and lower *T*_max_ estimates were observed in healthy volunteers as compared with patients with impaired pulmonary function. This is possibly a consequence of the differences in administration time of Lumason® between the two studies (approximately 15 s in healthy subjects versus approximately 20 s in patients). Half-life estimates were similar between the two studies with means of 11.6 and 9.9 min for patients and healthy volunteers, respectively. Comparison of AUC estimates indicate that the extent of exposure to Lumason® in healthy volunteers is approximately double the exposure achieved in patients with impaired pulmonary function, with mean values of 10.26 and 5.87 ng.min/ml, respectively. As AUC is used to calculate clearance, the lower values of AUC observed in patients with impaired pulmonary function may account for the higher mean estimate of apparent total body clearance in patients (20,520 L/hr) as compared with healthy volunteers (8,298 L/hr).

**Table 6:** Pharmacokinetic parameters of SF6 in patients with pulmonary impairment and healthy subjects following intravenous administration of Lumason® 0.3 ml/kg.

Parameter (blood)	Patients with pulmonary impairment^a^	Healthy subjects^b^
*C*_max_ (ng/ml)	1.45 ± 1.03^c^ (0.35–3.79)	3.53 ± 1.77 (1.29–7.79)
*T*_max_ (min)	2.2 ± 0.8 (1.0–4.0)	1.5 ± 0.5 (1.0–2.0)
AUC (ng.min/ml)^d^	5.87 ± 4.04 (1.49–15.08)	10.260 ± 3.354 (5.486–15.589)
CL/F (L/hr)	20,520 ± 14,232 (5,208–49,566)	8,298 ± 3,466 (3,514–15,699)
*t*½*_λ_*_z_ (min)	11.64 ± 9.23 (1.54–29.09)	9.88 ± 8.73 (1.88–32.95)
Parameter (expired air)		
Recovery (% of dose)	102.2 ± 18.4^a^ (69.7–128.7)	93.9 ± 27.4^a^ (61.0–153.4)

AUC_(0—∞)_, area under the blood-concentration time curve; *C*_max_, maximum blood concentration; *T*_max_, time of maximum blood concentration; CL/F, apparent total body clearance; *t*½_λz_, terminal elimination half-life; Vss/F, apparent volume of distribution at steady-state.

^a^ *N* = 12 patients with pulmonary impairment.

^b^ *N* = 12 healthy subjects.

^c^ Values presented are arithmetic mean ± standard deviation and range (minimum—maximum).

^d^ AUC is from time 0 to infinity for study in patients with pulmonary impairment and from time 0 to 60 min for study in healthy subjects.

The percent of dose recovered in expired air ranged from 70% to 129% with an overall mean value of 102% in DIPF patients. In comparison, the percent of dose recovered in healthy subjects ranged from approximately 61% to 153% with a mean of 93.9%. This finding indicates that patients with DIPF eliminate virtually all SF6 from Lumason® via their lungs rather than via an alternate elimination route, despite the impairment of lung function.

**Figure 4:**
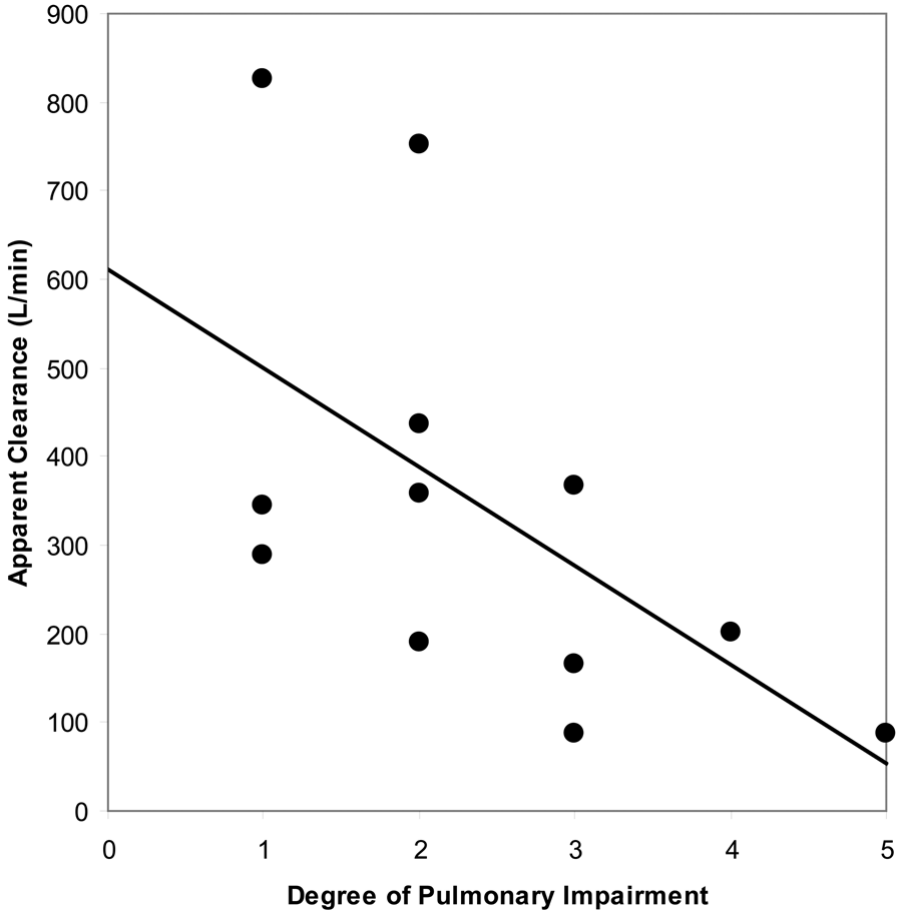
Relationship between apparent blood clearance of SF6 and degree of pulmonary impairment following intravenous administration of Lumason® 0.3 ml/kg to twelve patients with pulmonary impairment.

As shown in Figure 4, linear regression analysis demonstrated a statistically significant decrease in apparent total body clearance as the severity of pulmonary impairment increased (*p* = 0.0469). The strength of the relationship diminished when clearance estimates were normalized to weight (*p* = 0.0831).

No changes from baseline in vital signs or in oxygen saturation (<1%) up to 1 h post-dose were sufficient to meet the criteria for potential clinical significance. No serious adverse events were reported.

#### Safety of Lumason® in critically ill patients

Data on a total of 3,942 critically ill patients who had undergone either a Lumason®-enhanced echocardiogram (774 patients) or an unenhanced echocardiogram (3,168 patients) over the course of approximately 9 years were collected from 13 European hospitals. Ninety-eight of these patients did not meet the inclusion criteria; therefore, only 757 Lumason® patients and 3,087 control patients were included in the analysis population. Of the 757 patients who underwent a Lumason®-enhanced echocardiogram, 435 received an actual mean volume of 3.75 ml of Lumason® (range: 0.8–12.0 ml) while 198 received a “per hospital protocol” volume of 5.0 ml of Lumason®. The remaining 124 patients were administered an unknown volume of Lumason®. Upon further investigation into the amount of Lumason® reported as “unknown”, 9 out of 12 investigators declared that, overall, between 1 and 5 ml of Lumason® was administered per patient.

Of the 3,844 critically ill patients who met all the eligible criteria for the study, 53 (1.38%) died on the same day as the echocardiography procedure or on the following calendar day. Among these 53 patients, 48 (48/3,087 patients, 1.55%) had undergone unenhanced echocardiography, while 5 (5/757, 0.66%) had undergone a Lumason®-enhanced examination. Univariate analysis revealed no significant difference between these 2 groups (*p* = 0.067). The estimated crude odds ratio comparing the Lumason® group with the control group (0.42 with 95% CI: 0.17–1.06) implies that patients receiving Lumason® during echocardiography had a 58% lower risk of mortality on the same day or following day compared with patients who had undergone unenhanced echocardiography.

Propensity score matching was performed on more than 80% of Lumason® patients (615/757 patients) who could be matched 1-to-1 with control patients based on their closest baseline risk status.

Findings from propensity score matching were comparable to those from univariate analysis. Of the matched 615 patients who had undergone unenhanced echocardiography, 10 (1.63%) died within the same day as the echocardiography procedure or on the following calendar day. In comparison, only 5 (0.81%) of the 615 matched patients that received Lumason® during echocardiography, died on the same day or on the following calendar day. There was no significant difference between these 2 groups (*p* = 0.068, Table 7). The estimated adjusted odds ratio comparing the Lumason® Group with the control Group was 0.30 with 95% CI: 0.08–1.09 which implies that, given similar baseline characteristics and risk factors, patients receiving Lumason® during echocardiography had a 70% lower risk of mortality on the same day or on the following calendar day compared with patients who had undergone unenhanced echocardiography.

**Table 7:** Comparison of in-hospital mortality on the same day or on the day following echocardiography between Lumason-enhanced echocardiography and non-enhanced echocardiography in critically ill patients—propensity score matched analysis.

	Control group (*N* = 615)	Lumason group (*N* = 615)	OR^a^ (95%CI^b^)	*p*-value^c^
In-hospital mortality *n* (%)	10 (1.63%)	5 (0.81%)	0.30 (0.08, 1.09)	0.068

^a^ OR is the estimated adjusted odds ratio comparing Lumason Group versus Non-Contrast Group from conditional logistic regression model based on propensity score matched subjects.

^b^ 95% CI is 95% confidence interval for adjusted OR.

^c^ *p*-value is from Wald chi-square test of logistic regression analysis.

A supportive multivariate logistic regression analysis that included all eligible patients confirmed the results of the propensity score matched analysis. The adjusted odds ratio comparing the Lumason® Group with the control Group was 0.33 with 95% CI: 0.12–0.89 which implies that, after adjusting for baseline characteristics and risk factors, patients receiving Lumason® had a 67% reduction in the risk of mortality on the same day or on the following calendar day than patients who had undergone unenhanced echocardiography. This reduction was statistically significant (*p* = 0.028).

Analysis of the composite endpoint of mortality and major adverse events showed that death or major adverse events within the same day or on the following calendar day were recorded for 50 (1.62%) of the 3,087 patients who had undergone unenhanced echocardiography and 11 (1.45%) of the 757 patients that received Lumason® during echocardiography. The odds ratio comparing the Lumason® group with the control group was 0.90 with 95% CI: 0.47–1.71. There was no difference between these 2 groups (*p* = 0.8714).

#### Safety of Lumason® from post-marketing surveillance

A total of 3,182 cases [Reporting Rate (RR) of 0.0244%] which were considered possibly or probably related to the administration of Lumason® were spontaneously reported in approximately 13 million patients exposed to the agent during market use. Of these 3,182 cases, 1,166 were classified as serious (RR for serious adverse events of 0.0096%). The overall incidence of serious hypersensitivity reactions was 0.007% (<1 in 10,000; 69% of reported serious adverse events).

## Discussion

The studies presented herein provide a comprehensive overview of the safety and tolerability of Lumason® in special patient populations. The patients enrolled in these studies reflect the types of patients that would potentially benefit from the improved quality of images obtained with Lumason®-enhanced echocardiography.

### Effects of Lumason on ventricular repolarization in patients with CAD

Two studies were performed to evaluate the effects of Lumason® on ventricular repolarization in patients with CAD. Possible effects were assessed by acquiring continuous ECG recordings using a 12-lead Holter device. This approach permits the recording of electrical signals over a long period of time and can show dynamic changes in heart rate or rhythm, including circadian variations. These data can be analyzed for several parameters, including arrhythmias, heart rate variability, and QT interval (33).

In one of the two studies, the effects of Lumason® on QT interval was assessed during continuous heart insonation at both low (0.4–0.5) and high (1.5–1.6) MI. High MI has previously been considered a risk factor for arrhythmias based on pre-clinical studies that suggested that destruction of contrast microbubbles by high MI ultrasound exposure was associated capillary rupture and endothelial cell damage (34). Furthermore, a study conducted in humans with a non-commercial ultrasound contrast agent revealed an increase in premature ventricular contractions (PVCs) using insonation triggered on end-systole at a high MI of 1.5 (35).

Overall, the effect of Lumason® on QT interval in patients with documented CAD was similar to that of placebo, even at doses that were 3.5 and 17.5 times the recommended clinical dose for echocardiography (2 ml corresponding to 0.03 ml/kg in a 70-kg person) and with continuous insonation of the heart at high MI values up to 1.5–1.6. These results suggest that administration of Lumason® to patients with CAD is not associated with an increased risk of prolonged repolarization.

### Effects of Lumason® on pulmonary hemodynamics and cardiac function in patients with pulmonary hypertension or congestive heart failure

Although no negative effects on oxygen saturation or negative inotropic effects have been reported in previous clinical trials of Lumason®, safety studies were nevertheless conducted in patients with compromised pulmonary and cardiac function due to the fact that UEA microbubbles pass through the pulmonary vascular bed, potentially leading to pulmonary capillary obstruction.

No significant effects on pulmonary hemodynamics were seen with Lumason® either in patients with normal baseline mean pulmonary arterial pressure or in patients with elevated mean pulmonary arterial pressure. Individual changes from baseline were small and of similar amplitude to that seen with placebo. A slightly higher variability was observed in patients with pulmonary hypertension when compared to patients with normal pulmonary arterial pressure with both Lumason® and placebo. This higher variability likely reflects the known changes in pulmonary hemodynamics previously described in patients with pulmonary hypertension, which may occur spontaneously (36), and which have been reported in clinical trials performed with other UEAs (13, 17, 37, 38). In general, pulmonary hemodynamics in patients with pulmonary hypertension are not affected by the administration of UEAs.

### Impact of Lumason on oxygen saturation and pulmonary function in patients with COPD

The study in patients with moderate or severe COPD was designed to evaluate potential changes in pulmonary function through repeated measurements of FEV1, FVC, and FEF_25%−75%_. These parameters are considered to have good sensitivity for diagnosing even minimal airflow limitations (39, 40).

No effects of Lumason® were seen on pulmonary or cardiovascular function or oxygen saturation, and no adverse events were reported which would raise safety concerns. The effects of 4 ml of Lumason® were comparable to those observed following administration of the same volume of placebo. Based on these findings, an effect of Lumason® on pulmonary function in patients with COPD appears unlikely even at doses 2 times higher than the recommended clinical dose for a single administration.

### Effects of Lumason on pharmacokinetics and oxygen saturation in patients with DIPF

DIPF did not impede pulmonary elimination of SF6, as recovery of SF6 in expired air was similar across patients with varying degrees of pulmonary impairment and similar to that observed in healthy subjects. The percent of dose recovered in expired air ranged from 70% to 129% with an overall mean value of 102% in DIPF patients following administration of Lumason® at a dose of 0.3 ml/kg. In comparison, the percent of dose recovered in healthy subjects ranged from 61% to 153% with an overall mean value of 93.9%. Likewise, the blood clearance of Lumason® in patients with DIPF was consistent with findings observed in healthy patients. Notably, the apparent total body clearance of SF6 in patients with the most severe pulmonary impairment was not markedly lower than that observed in healthy subjects. Based on these results, no adjustment of Lumason® dose is necessary in patients with pulmonary fibrosis.

### Safety of Lumason® in critically ill patients

Several studies have evaluated the safety of UEAs in critically ill hospitalized patients undergoing clinically indicated echocardiography (7, 14, 19, 41). Conclusions were drawn based on short-term mortality (24–48 h) among patients undergoing UEA-enhanced echocardiography compared to patients undergoing unenhanced echocardiography. Kusnetzky et al. (40) compared acute mortality in all hospitalized patients undergoing echocardiography with Definity® (*n* = 6,196) with patients undergoing unenhanced echocardiography (*n* = 12,475). Patients who received Definity® exhibited higher clinical acuity and more comorbidity than patients undergoing unenhanced echocardiography. Nevertheless, even though more critically ill patients received the UEA, there was no increase in 24 h mortality among patients that received the UEA. In another study, Main et al. (7) compared 1-day mortality among 58,254 patients who underwent echocardiography with Definity® compared with that among 4,242,712 patients who underwent unenhanced echocardiography. Unadjusted mortality was similar for the two groups [1.06% mortality at 1 day in the UEA group vs. 1.08% in the unenhanced group (*p* = 0.613)]. Multivariate regression analysis adjusting for key baseline covariates revealed that patients who underwent Definity®-enhanced echocardiography were 24% less likely to die within 1 day as compared with patients who underwent unenhanced echocardiography (odds ratio = 0.76, 95% CI: 0.70 to 0.82). Similar conclusions were drawn by Exuzides et al. (14) who performed a retrospective case-control analysis of mortality in critically ill patients undergoing echocardiography with Optison® compared with matched control patients. No significant difference in mortality was observed in the contrast-enhanced echocardiography group compared with the unenhanced group (odds ratio = 1.18; 95% CI: 0.82 to1.71; *p* = 0.37). Finally, Main et al. (19) compared 48 h all-cause mortality, including hospital stay mortality, among critically ill patients who underwent echocardiography with Definity® (*n* = 16,222 patients) compared with that among critically ill patients who underwent unenhanced echocardiography (*n* = 990,159 patients). Patients undergoing contrast-enhanced echocardiography had lower mortality at 48 h compared with patients undergoing unenhanced echocardiography (1.70% vs. 2.50%), with an odds ratio of 0.66 [95% confidence interval (CI): 0.54 to 0.80]. Patients undergoing contrast-enhanced echocardiography also had lower hospital stay mortality compared with patients undergoing unenhanced echocardiography (14.85% vs. 15.66%), with an odds ratio of 0.89 (95% CI: 0.84–0.96).

The results of all these studies suggest that UEA-enhanced echocardiography is a safe and reliable technique in critically ill patients requiring echocardiography. The results of our study with Lumason® are comparable with results obtained with other UEAs (7, 14, 19, 41), further confirming the excellent safety profile of these agents. Specifically, in our study no statistically significant difference for in-hospital mortality was noted between critically ill patients undergoing Lumason®-enhanced echocardiography and critically ill patients undergoing unenhanced echocardiography.

### Safety of Lumason® in post-marketing surveillance

To provide additional insight into the safety and tolerability of Lumason®, we determined the RR of adverse events collected from post-marketing surveillance from over 2 decades of clinical use worldwide. The RR of serious and non-serious adverse events combined was 0.0244% while the RR for serious adverse events alone was 0.0096%. The overall incidence of serious hypersensitivity reactions (<1 in 10,000) is similar to incidences reported for other UEAs (9). These data indicate that the frequency of adverse events related to the administration of Lumason® is extremely low, and that hypersensitivity reactions are rare.

In conclusion, the results of these studies confirm that Lumason® is an appropriate UEA for key patient populations, including patients with compromised cardiopulmonary conditions and critically ill patients. Importantly, these groups of patients represent those that may benefit considerably from the use of UEAs to improve echocardiogram image quality, and thus evidence supporting the safe use of Lumason® in these patients is essential. Although some studies had relatively small patient numbers and may have evaluated different Lumason/Sonovue doses, the overriding conclusion from these studies is that Lumason® is a safe and effective UEA for use in patients with compromised cardiopulmonary conditions and critically ill patients.

## Data Availability

Raw data supporting the conclusions of this article are on file at Bracco and available upon request.

## Appendix—Detailed Methods

### Assessment of potential effects on ventricular repolarization in patients with coronary artery disease

Two prospective clinical pharmacology studies assessed possible predictable untoward side effects on ventricular repolarization after Lumason® administration in patients with documented coronary artery disease (CAD) undergoing echocardiography.

In one study (single-blind, placebo-controlled, randomized, 3-way crossover study), each subject received two bolus injections of Lumason® at doses of 0.1 and 0.5 ml/kg, and one injection of placebo (0.9% normal physiological saline) in a sequence determined by randomization. The volume of placebo administered was evenly distributed between 0.1 and 0.5 ml/kg within each sequence. To simulate the conditions under which Lumason® would be administered in a clinical setting, two-dimensional echocardiography of the left ventricle [mechanical index (MI) 0.7 to 0.8] was performed for all patients starting 30 s prior to each administration of study agent and continuing for 5 min after the conclusion of study agent administration.

In the second study (single-blind, placebo-controlled, randomized, 4-way crossover study), each subject received 2 injections of Lumason® and 2 injections of placebo (0.9% normal physiological saline) at the same dose of 0.1 ml/kg; two different MI settings (low/medium: 0.4–0.5 and high: 1.5–1.6) with continuous insonation were used for echocardiography with each of the Lumason® and placebo injections. Each subject received the four treatments (i.e., Lumason® 0.1 ml/kg at MI 0.4, Lumason® 0.1 ml/kg at MI 1.5, placebo 0.1 ml/kg at MI 0.4, and placebo 0.1 ml/kg at MI 1.5) according to predefined sequence randomization. Two-dimensional (2D) echocardiography of the left ventricle was performed starting 30 s before start of study agent administration and continued for approximately 20 min after the conclusion of study agent administration.

In both studies, the administration of each study agent (Lumason® or placebo) was separated by at least 48 h; an infusion rate of 6 ml/min was used. Continuous 12-lead ECG data were collected from 3 h predose to 12 h postdose following each administration of study agent. The ECG recordings were processed by a central laboratory. Manual digitization of 3 beats from Lead II for the RR, PR, QRS, and QT interval durations at protocol-specified time points was performed. Board-certified cardiologists, blinded to the identity of study agent, verified the interval duration measurements, and interpreted each ECG for presence of pathological U waves and clinically significant T wave changes.

Other safety assessments performed included evaluation of clinical and laboratory markers for potential microembolism, monitoring for the occurrence of adverse events, recording of vital signs and pulse oximetry, laboratory evaluations, physical examination, Mini Mental Status Examination (MMSE), and neurological examination. Safety was monitored for up to 48 h for the first study and 72 h for the second study after the last administration of study agent. Patients remained in the clinic for the duration of the study.

### Assessment of potential effects of Lumason® on pulmonary hemodynamics and cardiac function

#### Patients with pulmonary hypertension

A multi-center, double-blind, randomized, placebo-controlled, intra-subject crossover study evaluated the effect of intravenous bolus injections of Lumason® in comparison with placebo on pulmonary hemodynamics in patients with normal (<25 mmHg; normal group) or elevated (≥25 mmHg; hypertension group) baseline mean pulmonary arterial pressure who were scheduled to undergo right heart catheterization as part of their routine clinical evaluation.

Each subject received intravenously 1 bolus injection of 4.8 ml Lumason® and 1 bolus injection of 4.8 ml placebo (0.9% normal physiological saline) followed by a 5 ml saline flush. The order of Lumason® and placebo injections was randomized within each group. A minimum interval of at least 10 min was maintained between the 2 injections to allow enough time to acquire the required hemodynamic and other safety information.

All patients underwent a standard right heart catheterization using a Swan-Ganz catheter from a jugular or femoral venous access. The right atrial pressure and right ventricular pressure were measured once, when the catheter was introduced through the respective chamber before reaching the pulmonary artery; these measurements were collected as baseline clinical information. Multiple pulmonary hemodynamic and cardiac parameters were measured before and after investigational product administration. These parameters included systolic (PAPs), diastolic (PAPd), mean pulmonary artery pressures (MPAP), pulmonary capillary wedge pressure (PCWP), ECG, Oxygen saturation, heart rate (HR), and cardiac output (Qp). Pulmonary vascular resistance (PVR) was derived from MPAP, PCWP, and Qp, whereas stroke volume (SV) was derived from Qp and HR.

Measurements for PAPs, PAPd, and MPAP were obtained twice within 5 min prior to the first investigational product administration; the average of these measures was used as the baseline. After each injection of investigational product, the same measurements were obtained at 1, 4, 7 and 10 min. These pressure measurements were recorded for approximately 10 heartbeats at each time point. Measurements for PCWP, Qp and HR were recorded within 5 min prior to the first investigational product administration as the baseline and repeated at 1 and 10 min after each investigational product injection.

Monitoring for adverse events was also performed. Physical examination, clinical laboratory analyses and ECG were performed at baseline and at 24 h after the last administration of the investigational product (either Lumason® or placebo).

#### Patients with congestive heart failure

The effects of Lumason® on pulmonary hemodynamics and cardiac function were also assessed in patients with congestive heart failure [NYHA (New York Heart Association) class II-III] and ejection fraction (EF) <45% referred for right cardiac catheterization (31).

In this placebo-controlled, single center, randomized study, patients were randomized to one of two arms: one arm receiving two bolus injections of Lumason® (2.0 and 4.0 ml) and two injections of placebo (2.0 and 4.0 ml of 0.9% physiological saline) according to a four-dose sequence randomization (Lumason® and placebo were administered alternately); one arm receiving two injections of placebo (2.0 and 4.0 ml) only according to a two-dose sequence randomization. The interval time between injections was 15 min or until disappearance of contrast effect. In addition, before each study agent injection, it was required that hemodynamic parameters after previous injections had returned to baseline levels.

The effects of Lumason® compared with placebo on pulmonary hemodynamics and cardiac function were monitored by right heart catheterization before the first administration and again at 30 sec, 2, 4, 6, 10 min after each administration. The assessment was based on measurement of the following parameters: baseline pulmonary vascular resistance, mean pulmonary artery pressure, pulmonary capillary wedge pressure, right atrial pressure, cardiac output, stroke volume, systemic vascular resistance, systolic, diastolic and mean systemic blood pressure, heart rate, and oxygen saturation. Measurements were made by right heart catheterization before and 30 s, 2, 4, 6, 10 min after administration of Lumason®. In addition, 12-lead ECG recording, physical examination and clinical laboratory analyses were performed at baseline and at predefined timepoints post-dose up to 24 h after the last administration of the study agent. Monitoring for adverse events was also performed.

### Assessment of oxygen saturation and pulmonary function in patients with moderate to severe chronic obstructive pulmonary disease

A single center, single blind, cross-over, placebo controlled, intravenous fixed-dose study investigated the safety and tolerability of Lumason® in patients with moderate or severe chronic obstructive pulmonary disease. Each patient received a single administration of 4 ml of Lumason® and a single 4 ml administration of placebo in two separate sessions with a 48- to 72-hour period between injections. The pulmonary function tests, including forced vital capacity (FVC), forced expiratory volume (FEV_1_), and forced mid-expiratory flow (FEF_25%−75%_) were obtained at baseline, and at 1–3 min (FEV1 only) and 9–11 min after each injection. Additional FEV1 measurements were obtained at 30 min, 1 h, and 5 h after each injection. A decrease of 15% in FEV_1_ and of 340 ml in FVC defined substantial change in these parameters and were considered adequate for identifying clinically significant changes in both moderately and severely impaired patients (42, 43). Oxygen saturation was measured at 1 min intervals from 5 min pre-injection up to 15 min post-injection, using a finger-tip pulse oximeter.

Pre- and post-contrast physical examination, vital signs, blood oxygen saturation, 12-lead ECG, continuous ECG monitoring, clinical laboratory tests, and monitoring for adverse events was performed. Safety parameters were assessed at selected time points up to 24 ± 1 h following the last injection of study agent.

### Assessment of pharmacokinetics and oxygen saturation in patients with diffuse interstitial pulmonary fibrosis (DIPF)

An open-label, single dose, single center study was conducted to evaluate the pharmacokinetics and safety of a dose of 0.3 ml/kg Lumason® in patients with reduced alveolar function due to mild to severe DIPF associated with any autoimmune, industrial, occupational, infectious, or connective tissue disease, previously confirmed with appropriate specific methodologies. Comparison was made with the pharmacokinetics and safety of an identical dose of 0.3 ml/kg Lumason® administered to subjects with normal alveolar function (i.e., subjects without DIPF).

Collection of blood and expired air samples for analysis of SF6 was completed approximately 2 h after Lumason® administration. Blood SF6 concentrations were determined at −1 min prior to Lumason® administration and at 0.5, 1, 2, 3, 4, 6, 8, 12, 16, 20, 30, 45, and 60 min post-dose. Concentrations of SF6 in expired air were determined at −2 to −1 min pre-dose and at 0 to 0.5, 0.5 to 1, 1 to 2, 2 to 3, 3 to 4, 4 to 6, 6 to 8, 8 to 11, 11 to 15, 15 to 20, 20 to 30, 30 to 40, 40 to 50, and 50 to 60 min post-dose.

Blood pharmacokinetic parameters were determined using non-parametric analyses. Blood distribution half-life was determined using the method of residuals. The relationship between apparent blood clearance of SF6 and degree of pulmonary compromise was determined using linear regression analysis. The rate of pulmonary elimination of SF6 was analyzed non-parametrically.

Patients were monitored for safety (vital signs, oxygen saturation, ECG, clinical laboratory tests) up to 24 h after dosing.

### Safety of Lumason® in critically ill patients

A retrospective non-interventional post-authorization study compared in-hospital mortality (defined as death within the same day of the echocardiography procedure or death on the following calendar day) between critically ill patients who had undergone contrast echocardiography with Lumason® and critically ill patients who had undergone echocardiography without the use of a contrast agent.

Patients were defined as critically ill according to at least one of the unstable cardiopulmonary conditions listed as the admitting diagnosis that included worsening or clinically unstable heart failure (Class III/IV), recent acute cardiac syndrome or clinically unstable ischemic cardiac disease, recent coronary artery intervention within 7 days prior to the echocardiogram, severe rhythm disorders, other factors suggesting clinical instability, severe pulmonary hypertension (pulmonary artery pressure >90 mmHg), adult respiratory distress syndrome, emphysema and/or COPD.

Information on adverse events was collected whenever such information was available in patients' medical records. An adverse event was defined as any untoward medical occurrence that occurred immediately after the start of the echocardiography and within the same day and/or the following calendar day. A blinded medical review of all major adverse events reported was performed by 2 physicians to confirm major events of interest (e.g., new major cardiac events, worsening of cardiac condition, and hypersensitivity reactions).

Data analysis for the primary endpoint of in-hospital mortality was performed as follows:

– Univariate Analysis: The incidences of acute in-hospital mortality were estimated for each study group. The crude odds ratio with the 95% CI for comparison of acute in-hospital mortality between the 2 groups was derived from the univariate logistic regression model.
– Propensity Score Matched Analysis: This was used to reduce potential biases due to confounding factors in the estimation of treatment effect on the same day or the next day following echocardiography. The intent of propensity analysis mimicked the conditions of a randomized trial such that patients were similar in every measurable respect except for treatment (Lumason® or Control) allocation (44). To compare in-hospital same day or the following calendar day mortality between matched Lumason® and Control patients, a conditional logistic regression model was applied to estimate treatment effects. The propensity score was included in the final conditional logistic regression model. The adjusted odds ratio was estimated and presented along with 95% confidence intervals.
– Multivariate Analysis: A supportive multivariate logistic regression analysis was applied for the comparison between the 2 groups to confirm the results of the propensity score matched analysis.

Analysis of composite endpoint of mortality and major adverse events was also performed for each study group.

### Safety of Lumason® from post-marketing surveillance

The total number of serious and non-serious adverse events that were spontaneously reported during market use from 1 April 2001 to 28 February 2023 which were considered possibly or probably related to the administration of Lumason® were collected as part of post-marketing surveillance.

An adverse event was classified as “serious” if it, (1) required inpatient hospitalization or prolongation of existing hospitalization, (2) resulted in persistent or significant disability/incapacity (where disability was defined as a permanent or substantial disruption of ability to carry out normal life functions, either reported or defined as per clinical judgement), (3) was a congenital anomaly/birth defect, (4) resulted in death, (5) was life-threatening (i.e., the patient was at risk of death at the time of the event/reaction; it does not refer to an event/reaction which hypothetically might have caused death if it were more severe) or (6) was any other “important medical event”, i.e., may not result in death, be life-threatening, or require hospitalization, but, based upon appropriate medical judgment, it may jeopardize the subject and may require medical or surgical intervention to prevent any of the outcomes listed in the definition above.

Adverse events were classified as “non-serious” if the adverse event/reaction did not meet the criteria listed for a serious event.
